# Expanded profiling of Remdesivir as a broad-spectrum antiviral and low potential for interaction with other medications in vitro

**DOI:** 10.1038/s41598-023-29517-9

**Published:** 2023-02-23

**Authors:** Sheli R. Radoshitzky, Patrick Iversen, Xianghan Lu, Jing Zou, Suzanne J. F. Kaptein, Kelly S. Stuthman, Sean A. Van Tongeren, Jesse Steffens, Ruoyu Gong, Hoa Truong, Annapurna A. Sapre, Huiling Yang, Xiaodong Xie, Jia Jun Chia, Zhijuan J. Song, Stacey M. Leventhal, Josolyn Chan, Alex Shornikov, Xin Zhang, David Cowfer, Helen Yu, Travis Warren, Tomas Cihlar, Danielle P. Porter, Johan Neyts, Pei-Yong Shi, Jay Wells, John P. Bilello, Joy Y. Feng

**Affiliations:** 1 The Geneva Foundation, United States Army Medical Research Institute of Infectious Diseases (USAMRIID), Fort Detrick, Frederick, MD USA; 2 Division of Antivirals, Office of Infectious Diseases, Center for Drug Evaluation and Research, Food and Drug Administration, Silver Spring, MD USA; 3 Gilead Sciences, Inc, 333 Lakeside Drive, Foster City, CA USA; 4 Department of Biochemistry & Molecular Biology, Institute for Drug Discovery, Sealy Center for Structural Biology & Molecular Biophysics, University of Texas Medical Branch, Galveston, TX USA; 5 KU Leuven Department of Microbiology, Immunology and Transplantation, Laboratory of Virology and Chemotherapy, Rega Institute for Medical Research, B-3000 Leuven, Belgium; 6 Global Virus Network, GVN, Baltimore, USA; 7 Laulima Government Solutions, LLC., Orlando, FL USA

**Keywords:** Drug discovery, Microbiology, Diseases, Pathogenesis

## Abstract

Remdesivir (GS-5734; VEKLURY) is a single diastereomer monophosphoramidate prodrug of an adenosine analog (GS-441524). Remdesivir is taken up by target cells and metabolized in multiple steps to form the active nucleoside triphosphate (GS-443902), which acts as a potent inhibitor of viral RNA-dependent RNA polymerases. Remdesivir and GS-441524 have antiviral activity against multiple RNA viruses. Here, we expand the evaluation of remdesivir’s antiviral activity to members of the families *Flaviviridae*, *Picornaviridae*, *Filoviridae*, *Orthomyxoviridae*, and *Hepadnaviridae.* Using cell-based assays, we show that remdesivir can inhibit infection of flaviviruses (such as dengue 1–4, West Nile, yellow fever, Zika viruses), picornaviruses (such as enterovirus and rhinovirus), and filoviruses (such as various Ebola, Marburg, and Sudan virus isolates, including novel geographic isolates), but is ineffective or is significantly less effective against orthomyxoviruses (influenza A and B viruses), or hepadnaviruses B, D, and E. In addition, remdesivir shows no antagonistic effect when combined with favipiravir, another broadly acting antiviral nucleoside analog, and has minimal interaction with a panel of concomitant medications. Our data further support remdesivir as a broad-spectrum antiviral agent that has the potential to address multiple unmet medical needs, including those related to antiviral pandemic preparedness.

## Introduction

Remdesivir (RDV; GS-5734; VEKLURY), the first FDA-approved antiviral to treat COVID-19, is a single diastereomer monophosphoramidate prodrug of an adenosine analog (GS-441524). Once taken up by cells, RDV is metabolized in multiple steps to form the active nucleoside 5′-triphosphate (TP), a potent inhibitor of multiple viral RNA-dependent RNA polymerases. RDV has broad-spectrum activity against many RNA viruses in cell culture, including coronaviruses (SARS-CoV-2, SARS-CoV, and MERS-CoV)^1–7^, picornaviruses (enterovirus 71 [EV71] and coxsackievirus B3)^8^, filoviruses (Ebola virus [EBOV], Sudan virus [SUDV], Bundibugyo virus, Marburg virus [MARV])^9–11^, pneumoviruses (respiratory syncytial virus [RSV])^10–12^, and paramyxoviruses (Nipah virus [NiV], measles virus, and Hendra virus)^13,14^. RDV has moderate activity against Lassa virus and Junin virus in the *Arenaviridae* family, and against tick-borne Alkhurma hemorrhagic fever virus, Kyasanur forest disease virus, Omsk hemorrhagic fever virus, and tick-borne encephalitis virus in the *Flaviviridae* family^10,13^. RDV has minimal antiviral activity against chikungunya virus and Venezuelan equine encephalitis virus in the *Togaviridae* family, human immunodeficiency virus type 1 (HIV-1) in the *Retroviridae* family, Rift Valley fever virus in the *Phenuiviridae* family, Crimean-Congo hemorrhagic fever virus in the *Nairoviridae* family, and vesicular stomatitis virus in the *Rhabdoviridae* family^10,13^. The reasons for variations in the activity profile are unknown, but likely reflect subtle differences at the active site of viral RNA-dependent RNA polymerases^15^.

In this study, we expanded the evaluation of RDV’s antiviral activity to other members of the families *Flaviviridae*, *Picornaviridae*, *Filoviridae* (with new strains), *Orthomyxoviridae*, and *Hepadnaviridae*. For pandemic preparedness, in the case multiple drugs might need to be combined to enhance antiviral activity and consequently efficacy, we also show a lack of antagonism between RDV and favipiravir, another approved broad-antiviral nucleoside analog, in antiviral assays against two representative filoviruses. In addition, we demonstrate that there is no antagonsim between RDV and a panel of concomitant medications commonly used in SUDV- and MARV-endemic regions.

## Results

### Remdesivir potency against respiratory viruses varies based on virus family

RDV potency against RSV and various coronaviruses in vitro has been extensively profiled^1,3–7,12^. We confirm that RDV is potent against the endemic OC43 and 229 E coronavirus, with an EC_50_ value of 0.067 µM in Huh7 cells and 0.093 µM in H1 HeLa cells respectively (Table 1). In cell-based infection assays, RDV inhibited enteroviruses 68D and 71 with EC_50_ values of 0.050 and 0.140 μM, respectively. The potency of RDV against rhinoviruses of serotypes A and B ranged from EC_50_ values of 0.385 to 0.750 μM in H1 HeLa cells. Conversely, RDV was inactive against influenza A and B (EC_50_ > 50 μM; Table 1).

**Table 1:** Antiviral activity of RDV against a broad panel of infectious human viruses.

Viral family	Virus (strain) [cells]	Antiviral activity EC_50_ (µM)^a^	CC_50_ (µM)	Selectivity index (SI)^b^	Previously reported activity EC_50_ (µM)
*Corona-*	OC43 [Huh-7]	0.067 ± 0.005	18.9 ± 1.8	282	0.096–0.15^16,17^
	229E [H1 HeLa]	0.093 ± 0.053	> 50	> 538	0.024^18^
*Picorna-*	Enterovirus 68D [RD]	0.050 (n = 1)	2.82	56	0.1–1^8^
	Enterovirus 71 [RD]	0.140 (n = 1)	3.31	24	NA
	Rhinovirus A2 [H1 HeLa]	0.450 (n = 1)	> 10	> 22	NA
	Rhinovirus 14 [H1 HeLa]	0.385 ± 0.318		> 26	
	Rhinovirus 16 [H1 HeLa]	0.750 (n = 1)		> 13	
*Flavi-*	Dengue 1 virus (Western Pacific) [Huh-7]	0.21 (n = 1)	33.4	159	NA
	Dengue 1 virus (Djibouti) [Huh-7]	0.31 ± 0.09		108	
	Dengue 2 virus (New Guinea C) [Huh-7]	0.23 (n = 1)		145	
	Dengue 2 virus (RL) [Huh-7]	0.29 ± 0.07		115	
	Dengue 3 virus (VN32) [Huh-7]	0.12 (n = 1)		278	
	Dengue 3 virus (H87) [Huh-7]	0.17 ± 0.07		196	
	Dengue 4 virus (MY01) [Huh-7]	0.18 (n = 1)		186	
	Dengue 4 virus (Dakar_HD_34460) [Huh-7]	0.19 ± 0.05		176	
	Zika virus (PRVABC59) [Huh-7]	0.26 (n = 1)	33.4	128	NA
	Zika virus (Dakar) [Huh-7]	0.37 (n = 1)		90	
	Zika virus (MR766) [Huh-7]	1.15 ± 0.03		29	
	Yellow fever virus (YFS11) [Huh-7]	1.06 (n = 1)	33.4	32	NA
	Yellow fever virus (17D) [Huh-7]	0.18 ± 0.03		186	
	Japanese encephalitis (14–14-2) [Huh-7]	0.34 (n = 1)	33.4	98	NA
	West Nile (NY99) [Huh-7]	0.05 (n = 1)	33.4	668	NA
*Filo-*	Ebola virus (Makona) [HeLa]	0.078 ± 0.004	> 2.5	> 32	0.003–0.14^10,13^
	Ebola virus (Mayinga) [Hela]	0.136 ± 0.017		> 18	
	Ebola virus (Kikwit) [HeLa]	0.070 ± 0.008		> 36	
	Bundibugyo virus [Hela]	0.19 ± 0.02	> 10	> 53	NA
	Sudan virus (Gulu)[HeLa]	0.06 (n = 1)	> 11.5	> 192	NA
	Sudan virus (Boneface) [HeLa]	0.12 (n = 1)	> 2.5	> 21	
	Sudan virus (Yambio) [HeLa]	0.13 (n = 1)	> 2.5	> 19	
	Marburg virus (Ci67) [HeLa]	0.030 ± 0.002	> 2.5	> 83	0.01–0.02^13^
	Marburg (Musoke) [HeLa]	0.04 ± 0.004	> 2.5	> 63	NA
	Marburg (Kabale) [HeLa]	0.04 ± 0.002	> 2.5	> 63	NA
	Marburg (Nganda) [HeLa]	0.024 ± 0.004	> 2.5	> 104	NA
	Marburg (Drandema) [HeLa]	0.034 ± 0.004	> 2.5	> 74	NA
	Marburg (Angola) [HeLa]	0.045 ± 0.03	> 2.5	> 56	NA
	Ravn Virus [HeLa]	0.068 ± 0.01	> 2.5	> 37	NA
*Hepaci-*	Hepatitis C (1b) [Huh-7-lunet], replicon	0.089 ± 0.047	> 20	> 225	0.057^11^
	Hepatitis C (2a) [Huh-7-lunet], replicon	0.072 ± 0.013	17 ± 3	236	NA
*Orthomyxo-*	Influenza A (A/California/07/2009) [MDCK]	> 50	> 100	Low	NA
	Influenza B (B/Brisbane/60/2008) [MDCK]	>50		Low	
*Hepadna-*	Hepatitis B (HepG2)	3.9 to > 10	7.4	Low	NA
	Hepatitis B (PHH)	> 1	> 1	Low	
	Hepatitis D (Huh7-NTCP)	> 5	8–10	Low	NA
	Hepatitis E GT3 Kernow C1 p6/Luc replicon [Huh-7]	> 2.07	2.07	Low	NA

*NA* data is not available.

^a^Unless noted, EC_50_ values are average ± standard deviation from at least 2–3 independent measurements. The studies marked n = 1 contain 2–4 technical replicates. For ease of comparison, literature values are listed when applicable. However, different cells and/or different assay protocols were used to generate the literature values.

^b^Selectivity Index = CC_50_/EC_50_. When minimal RDV antiviral activity is observed for a particular virus, low SI is indicated.

### Remdesivir is a potent inhibitor of pathogenic flaviviruses

The activities of GS-441524 against dengue virus-2 (DENV-2), yellow fever virus (YFV), and West Niles virus (WNV) have been reported to be EC_50_ of 9.46, 11, and > 30 µM, respectively^19^. In this study, RDV activity against flaviviruses was evaluated in cell-based infectious assays. Huh-7 cells were treated with increasing concentrations of compound and subsequently exposed to DENV 1–4, zika virus (ZIKV), YFV, or Japanese encephalitis virus (JEV) expressing a nano luciferase reporter protein. Luciferase activity was measured at the assay endpoint as an output of virus infection. Cell viability was tested on the same cell type using ATP-based luminescent assay to measure cytotoxicity. RDV reduced infection by all of these flaviviruses, with highest potency against DENV (EC_50_ range = 0.12–0.23 μM; Table 1) and lowest potency against YFV (EC_50_ = 1.06 μM; Table 1). RDV antiviral activity against WNV was evaluated in a similar manner with the exception that virus infection rates were measured via plaque assays of culture media at the assay endpoint. RDV was a potent inhibitor of WNV infection (EC_50_ = 0.05 μM; Table 1).

### Remdesivir is a potent inhibitor of various MARV and SUDV variants/isolates

RDV antiviral activity of RDV against filoviruses has been previously reported in both in vitro and in vivo models of infection^9–11,13,20^. However, these studies focused on filoviruses such as EBOV variants Kikwit, Makona, and Yambuku (isolate Mayinga), SUDV variant Gulu, and MARV variants Angola and Hesse (isolate Cieplik aka “Ci67”) (summarized in Table 1). To examine RDV’s antiviral activity against geographically and temporally distinct filovirus isolates, dose–response studies were performed in human HeLa cells, and virus infection rates were determined based on viral antigen staining at the assay endpoint^10,21,22^. Cell viability was evaluated simultaneously by nuclei and cytoplasm staining of assay wells. As summarized in Table 1, RDV was a potent inhibitor of all MARV and Ravn virus (RAVV) isolates tested with EC_50_ values 0.024–0.068 μM. Strong, albeit less potent, activity was also observed against all SUDV isolates tested (EC_50_ values of 0.12–0.24 μM).

### Remdesivir is a potent inhibitor of HCV replicon

Early studies showed GS-441524 and RDV is active against HCV 1b replicon with EC_50_ of 3.1 µM and 0.057 µM, respectively^11,19^. In this study, we evaluated RDV activity against both HCV genotype 1b and 2a using a subgenomic replicon system in which Renilla luciferase reporter levels serve as a measure of virus replication. RDV inhibited replicon activity of both HCV genotypes with similar efficacy (EC_50_ = 0.072–0.089 μM; Table 1).

### Remdesivir is a poor inhibitor of hepatitis B, D, and E viruses

RDV was tested for antiviral activity against hepatitis B virus (HBV; AD38 strain) in HepG2 or PHH cells. The observed potency of RDV was similar to the CC_50_ of the compound in these cells. Therefore, the selectivity index of RDV against HBV is near 1, suggesting the cytotoxicity of RDV is the likely cause of the measured antiviral effect in our assays. RDV is inactive against HDV in Huh-7 cells with a EC_50_ > 5 µM. In addition, RDV was inactive against hepatitis E virus (GT3-Kernow C1 p6/Luciferase replicon) at concentrations up to 1 µM. Higher concentrations were not attempted as cytotoxicity was observed at concentrations of approximately 4 µM.

### Combination of RDV and favipiravir against SUDV and MARV infection in cell-based assays

Favipiravir (Avigan, T-705) is a nucleoside analog with broad-spectrum activity against RNA viruses such as arena-, bunya-, nairo-, flavi-, and filoviruses^23,24^. It was approved in Japan in 2014 but was restricted to treat novel or re-emerging influenza virus infections (not seasonal influenza) against which other influenza antiviral drugs are ineffective^25,26^.

Under its approved dose (1600 mg BID on Day 1; 600 mg BID on Days 2–5), favipiravir showed a time-dependent decrease in exposure after continuous use in both healthy volunteers^27,28^ and Ebola virus disease patients^29^. In the 2014–2015 JIKI trial (an experimental treatment with favipiravir for Ebola virus disease), a higher dose (2400 mg + 2400 mg + 1200 mg on Day 0; 1200 mg BID on Days 1–9) produced a plasma median concentration of 46.1 μg/mL (~ 310 μM) on Day 2, with an ~ 50% decrease on Day 4 (25.9 ug/mL, ~ 170 μM)^29^. In our cell-based infection assay, favipiravir showed low antiviral activity against SUDV and MARV, with EC_50_ values of 507 ± 79.9 and 113 ± 9.8 µM, respectively (Table S1 and S2). We also evaluated RDV’s activities in combination with clinically relevant favipiravir concentrations (23–750 μM) against SUDV (Table S1) and MARV (Table S2) infection. The EC_50_ of RDV was reduced in a dose-dependent manner in the presence of favipiravir (Table S1 and S2; maximum of ≥ 5 2- or ≥ tenfold reduction for SUDV and MARV, respectively). The effect of the compound combination on the EC_50_ of favipiravir was similar (Table S1 and S2; maximum of ≥ 21- or ≥ 4.8-fold reduction for SUDV and MARV, respectively). The median CI for the RDV and favipiravir combination was 1.12 for SUDV and 1.02 for MARV (Table 2). Thus, overall, the combination of these two compounds appears to have simple additive, and not antagonistic, antiviral effect against SUDV and MARV. To minimize software bias, we also used SynergyFinder (version 3) to analyze the combination of RDV + favipiravir for its anti-MARV activity^30^ (https://synergyfinder.fimm.fi). The software produced a synergy score of 1.965, suggesting the combination is additive based on the conventional cut-off of synergy scores: > 10 indicates synergy, − 10 to + 10 indicates additivity, and > 10 indicates antagonism (Fig. S1). The combination of the compounds showed no evidence of cytotoxicity, even at the highest concentrations of both compounds.

**Table 2:** Combination of RDV and favipiravir has additive effect on the antiviral activity against SUDV and MARV infections in cells, as shown by the combination index (CI), excess volume, and synergy score values.

Viruses	Minimum CI	Median CI	Maximum CI	Synergy model	Excess volume	Synergy score
SUDV	0.57	1.12	1.34	Loewe Model	− 0.52	− 0.06
				HSA Model	0.72	0.57
				Bliss Model	− 0.78	− 0.23
MARV	0.88	1.02	1.09	Loewe Model	− 0.45	− 0.29
				HSA Model	0.87	0.53
				Bliss Model	0.02	− 0.08

*CI* combination index, *HSA* highest single agent.

### Remdesivir interactions with concomitant medications

Eighteen therapeutic agents commonly used in SUDV- and MARV-endemic regions, including HIV antiviral therapies, antimalarial drugs, and medications used to ameliorate symptom of viral hemorrhagic fevers (Table S3, WHO 2010, 2016) were evaluated for potential interactions with RDV. All drugs were tested at their corresponding human plasma maximum concentration (C_max_) unless substantial toxicity was observed, which then led to tests at a lower concentration. As summarized in Table 3, the drugs are divided into two categories: (1) those with no antiviral activities against SUDV and MARV when tested alone. The majority of these drugs showed minimal effect on RDV potency in combination studies, including acetaminophen, artemether, atovaquone, diazepam, metronidazole, omeprazole, ondansetron, proguanil, lamivudine, ritonavir, and tenofovir disoproxil fumarate (TDF); whereas amodiaquine and ciprofloxacin enhanced RDV’s activity against SUDV by lowering the EC_50_ > 5-folds; (2) drugs that showed antiviral activity against SUDV (efavirenz, lopinavir, lumefantrine, and ceftriaxone were tested alone, or in combination with RDV (Table 3). None of these drugs showed antagonistic effect when tested against SARS-CoV-2 in combination with RDV. Enhanced cytotoxicity was observed for combinations of RDV with efavirenz and atovaquone at 41 and 22.6 µM, respectively. These compounds had similar cytotoxicity when tested alone (CC_50_ ≈ 14–28 µM for efavirenz and CC_50_ ≈ 8–17 µM for atovaquone).

**Table 3:** Potency of RDV with concomitant medications commonly used in SUDV- and MARV-endemic regions tested in in vitro assays.

Category 1: Drugs with no innate activity against SUVD or MARV when tested in combination with RDV					
Drug of interest (tested at fixed concentration^1^)	RDV activity against SUDV when in combination		RDV activity against MARV when combination		Drug-drug interaction? (RDV potency)
	EC_50_^2^ (µM)	CC_50_ (µM)	EC_50_ (µM)	CC_50_ (µM)	
RDV alone	0.308 ± 0.313 (n = 9; 0.089, 0.936)	> 2.5	0.109 ± 0.054 (n = 9; 0.037, 0.189)	> 2.5	
Acetaminophen (75 μM)	0.50 ± 0.56	> 2.5	0.13 ± 0.02	> 2.5	No
Amodiaquine (0.07 μM)	0.052 ± 0.044	> 2.5	0.33 ± 0.10	> 2.5	Enhanced^5^ for SUDV
Artemether (0.7 μM)	0.48 ± 0.55	> 2.5	0.13 ± 0.03	> 2.5	No
Artesunate (1.2 µM)	0.31 ± 0.45	> 2.5	0.15 ± 0.03	> 2.5	No
Artesunate (8.5 µM)	< 0.02 (tox^3^)	< 0.2	0.16 ± 0.06	> 2.5	
Atovaquone (3.7 µM)	0.094 ± 0.067	> 2.5	0.08 ± 0.02	> 2.5	No
Atovaquone (22.6 µM)	< 0.02 (tox^3^)	< 0.02	0.016 ± 0.006	< 0.02	
Ciprofloxacin (14 µM)	0.041 ± 0.002	> 2.5	0.14 ± 0.03	> 2.5	Enhanced for SUDV
Diazepam (1.7 µM)	0.50 ± 0.49	> 2.5	0.11 ± 0.04	> 2.5	No
Metronidazole (30 µM)	0.42 ± 0.46	> 2.5	0.103 ± 0.005	> 2.5	No
Omeprazole (3.2 µM^4^)	0.22 ± 0.19	> 2.5	0.15 ± 0.07	> 2.5	No
Ondansetron (0.3 µM)	0.40 ± 0.33	> 2.5	0.12 ± 0.03	> 2.5	No
Proguanil (3.2 µM)	0.57 ± 0.59	> 2.5	0.16 ± 0.05	> 2.5	No
Lamivudine (11.3 µM)	0.67 ± 0.75	> 2.5	0.14 ± 0.06	> 2.5	No
Lopinavir (15.6 µM)	See data below	See data below	0.29 ± 0.20	> 2.5	No for MARV
Ritonavir (1.8 µM)	0.35 ± 0.34	> 2.5	0.15 ± 0.05	> 2.5	No
Tenofovir disoproxil fumarate (0.5 µM)	0.10 ± 0.04	> 2.5	0.09 ± 0.03	> 2.5	No

Category 2: Drugs with innate activity against SUDV or MARV when tested alone					
Drug of interest	Activity against SUDV		Activity against MARV		
	EC_50_ (µM)	CC_50_ (µM)	EC_50_ (µM)	CC_50_ (µM)	
Efavirenz	2.24 ± 0.57	20.7 ± 10.1	19.8 ± 0.1	15.1 ± 4.1	
Lopinavir	2.32 ± 0.58	34.1 ± 0.9	> 100	> 100	
Lumefantrine	9.60 ± 0.52	40.0 ± 1.2	16.6 ± 7.3	37.3 ± 0.9	
Ceftriaxone	2.24 ± 0.57	20.9 ± 10.1	19.8 ± 0.1	15.1 ± 4.1	

Drugs from Category 2 against SUDV or MARV when tested in combination with RDV					
Drug of interest (tested at fixed concentration^1^)	RDV activity against SUDV when in combination		RDV activity against MARV when combination		Drug-drug interaction? (RDV potency)
	EC_50_^2^ (µM)	CC_50_ (µM)	EC_50_ (µM)	CC_50_ (µM)	
Efavirenz (5 µM)	< 0.015	> 2.5	0.13 ± 0.13	> 2.5	Not antagonistic
Efavirenz (11 µM)	ND	ND	0.075 ± 0.042	> 2.5	
Efavirenz (41 µM)	< 0.02	< 0.02	< 0.02	< 0.02	
Lopinavir (15.6 µM)	< 0.02	> 2.5	0.29 ± 0.20	> 2.5	Not antagonistic
Lumefantrine (12 µM)	0.026 ± 0.021	> 2.5	0.103 ± 0.005	> 2.5	Not antagonistic
Ceftriaxone (440 µM)	< 0.017	> 2.5	0.078 ± 0.011	> 2.5	Not antagonistic

Compounds are divided into two categories based on whether they have antiviral effects against SUDV or MARV as a single agent.

*ND* not determined.

^1^Concentration equivalent to human plasma C_max_ and has not been adjusted with protein-binding. Lower concentrations were used when significant cell killing was observed.

^2^EC_50_ values represent averages from 2–3 independent experiments.

^3^The observed antiviral effect is driven by cytotoxicity.

^4^Tested at concentration ~ 19-fold over reported C_max_ (unadjusted for protein-binding).

^5^Enhanced activity is defined by > fivefold increase of antiviral activity of the combination over the RDV-alone treatment.

## Discussion

In 1998, Barrett and his fellow anthropologist at Emory University published their theory on the upsurge of emerging infectious diseases since the late 1970’s including the HIV AIDS epidemic^31^. They proposed three epidemiologic transitions in human history defined by a unique pattern of diseases related to subsistence and social structure at the time, each associated with a rise or major shift in the impact of infectious diseases: (1) the first transition coincided with the Neolithic Revolution, where many of the contemporary human infections can be traced to the zoonoses of domesticated animals; (2) the second transition occurred during the Industrial Revolution in mid-nineteenth century Europe and North America, where developed countries experienced a marked decline in infectious disease mortality and a concomitant rise in non-communicable diseases such as degenerative, metabolic, and aging-related diseases; (3) the third transition was noted in the 1970’s for newly emerging pathogens tracked by the Centers for Disease Control and Prevention (CDC)^32^, including newly emerging, re-emerging, and drug-resistant viruses. Factors contributing to the emergence of new viruses or re-immergence of viruses, include climate change, ecological disruption, food animal industry, globalization, and public health system failures^31,33^. The COVID-19 pandemic reinforces the concept that future viral infections may have a global impact in a short period of time, making it critical to have antivirals with broad-spectrum activity^34^. The past 2 years have also highlighted the challenges of screening compound libraries to identify existing drugs for a new “repurposed” indication in the midst of a pandemic, while trying to shortcut or circumvent the process of quality lead identification and full optimization of small molecule antiviral candidates^35^.

In this study, we confirmed the broad-spectrum antiviral activity of RDV against human pathogenic RNA viruses and demonstrated that RDV is also a potent inhibitor of picornaviruses, pathogenic flaviviruses, and endemic coronaviruses. The observed RDV antiviral potency against additional flaviviruses and filoviruses is in accordance with prior studies with members of those viral families^10,13^. Antiviral activity has been observed in multiple cell types with different endpoint outputs: viral antigen expression, viral replication, viral genome copy number, cytopathic effect, and reporter gene expression. Members of viral families with little or no sensitivity to RDV include the orthomyxoviruses (influenza A and B), nairoviruses (Crimean Congo hemorrhagic fever virus), phenuiviruses (Rift Valley fever virus), togaviruses (chikungunya virus and the alphaviruses), and rhabdoviruses (vesicular stomatitis virus). Compared to its parent nucleoside GS-441524, RDV showed more potent antiviral activity against HCV, DENV-2, YFV, and WNV in cell-based assays^19^, likely related to the higher level of the 5’-triphosphate active metabolite formed in cells^3^.

In addition to treatment of natural viral infections, there is a need to develop broad-spectrum antiviral countermeasures for viruses that might be misused as warfare or bioterrorism agents^36^. A combination of two or more broad-spectrum antivirals is advantageous for protection from unknown pathogens. In this study, we showed that when RDV is combined with the broad-spectrum antiviral favipiravir, they have an additive effect, which makes the RDV + favipiravir combination a potential option in biodefense scenarios.

By cellular assays, RDV has low potential for antagonism with other concomitant medications; however, our results are limited to in vitro setting. Any potential in vivo drug-drug interactions should be assessed separately since the pharmacokinetics of each drug and its metabolite could be affected by many host factors beyond the scope of this study. As of April 2022, RDV is approved by the FDA for the treatment of SARS-CoV-2 in adults and children 28 days of age or older and weighing ≥ 3 kg^37,38^. The established antiviral potency, clinical safety, and corresponding dose regimen as an approved treatment for COVID-19 should facilitate further in vivo and clinical testing of RDV against a broader spectrum of RNA viruses indicated in this report. Ongoing in vivo assessments of RDV against emerging viruses^9,14^ and efficacy of orally available prodrug of the RDV parental nucleoside may support expanding the treatment options for neglected and emerging viral infections, and could potentially help addressing multiple unmet medical needs in infectious diseases including future antiviral pandemic preparedness^39,40^.

## Materials and methods

### Reagents and cell culture

Remdesivir was synthesized by Gilead Sciences, Inc. Favipiravir (6-fluoro-3-oxo-3,4-dihydropyrazine-2-carboxamide; T-705) was obtained from Fujifilm Toymana Chemical (Tokyo, Japan). E864-0360 was purchased from ChemDiv (San Diego, CA).

Human cervical carcinoma (HeLa) (ATCC, Manassas, VA, Cat#CCL-2) cells were cultured for 3 days in T715 or T225 tissue culture flasks in Minimum Essential Medium (MEM) (Corning, Corning, NY, Cat#10-009) supplemented with 10% fetal bovine serum (FBS) (Hyclone Laboritories/GE Health Care Life Sciences, Boston, MA) , 10% L-glutamine (Hyclone Laboritories), 10 mM HEPES (pH 7.0–7.6) (Sigma-Aldrich, St. Louis, MO), 1% non-essential amino acids (NEAA) (Sigma-Aldrich), 1% penicillin/streptomycin (Sigma-Aldrich). To generate assay plates, HeLa cell were seeded at 2,000 cells per well (in total of 40 μL per well) in imaging 384-well assay plates (Aurora Biotechnologies, Carlsbad, CA, Aurora 384, IQ-EB, 384 IQ-EB/NB, 200mclear, Cat#1052-11130) and incubated in a tissue culture incubator for approximately 20 h prior to compound treatment.

Rhabdomyosarcoma cells (RD; CCL-136), H1 HeLa (CRL-1958), and Madin-Darby canine kidney (MDCK; CCL-34) cells were obtained from ATCC. All cell lines were grown in either Eagle’s Minimum Essential Medium (EMEM) (Gibco/Thermo Fisher Scientific) or Dulbecco’s Modified Eagle’s Medium (DMEM) (Gibco/Thermo Fisher Scientific) supplemented with 10% FBS, 2.0 mM L-Glutamine, 100 units/mL penicillin and 100 µg/mL streptomycin. Cells were sub-cultured twice a week at a split ratio of 1:5 to 1:10 using standard cell culture techniques. Total cell number and percent viability determinations were performed using a hemocytometer and trypan blue dye-exclusion. Cell viability was greater than 95% for the cells to be utilized in the assays. The cells were seeded in 96-well tissue culture plates the day before the assay. Antiviral assays were performed at a reduced FBS concentration of 2%.

Human adenocarcinoma H1 HeLa (ATCC, Manassas, VA, Cat# CRL-1958) cell lines were maintained in high glucose DMEM (Gibco/Thermo Fisher Scientific) supplemented with 10% FBS (v/v).

Vero cells and Huh-7 cells were purchased from the America Type Culture Collection (ATCC, Bethesda, MD). Vero cells were maintained in high-glucose DMEM containing 10% FBS (HyClone Laboratories) and 1% penicillin/streptomycin (Invitrogen, Carlsbad, CA) at 37 °C with 5% CO_2_. Huh-7 cells were maintained in high-glucose DMEM supplemented with 4 mM GlutaMAX, 110 mg/L sodium pyruvate, 1% NEAA, 10% FBS and 1% penicillin/streptomycin. All culture medium, supplements and antibiotics were purchased from Thermo Fisher Scientific (Waltham, MA).

### Antiviral assay and corresponding cytotoxicity evaluation

The multiplicity of infection (MOI) used in the *in intro* antiviral assays are summarized in Table S4.

#### Coronavirus OC43 and the corresponding cytotoxicity assay

Human Coronavirus OC43 (HCoV-OC43) anti-viral assay was an anti-OC43 nucleoprotein ELISA. Specifically, Huh-7 cells were plated in 96-well plates (Corning Life Sciences, Durham, NA) at 0.1 mL/well at 0.144 × 10^6^ cells/mL in growth medium. After the cells were incubated at 37 °C in a humid atmosphere containing 5% (v/v) CO_2_ overnight, 0.1 mL per well OC43 virus diluted with the growth medium to 1.103 × 10^4^ pfu/mL was added to each well along with serial diluted test compounds dispensed by a HP D300e digital dispenser (Hewlett Packard, Palo Alto, CA) with a final volume of 0.2 mL/well. The culture was maintained in a humidified chamber at 33 °C with 5% (v/v) CO_2_ for 72 h. After the incubation, the culture media were removed, and the cells were fixed with 100% methanol for 10–15 min at room temperature. The plates were air dried for ten minutes after the fixative was removed. Afterward, the plates were blocked with 0.1 mL/well 10% FBS (Hyclone Laboritories), 5% dry milk (AmericanBio, Canton, MA), 0.1% Tween 20 (EMD Millipore, Burlington, MA) in PBS (Blocking buffer) for 30 min at 37 °C. After the blocking buffer was removed, anti-OC43 nucleoprotein antibody (EMD Millipore) were diluted 2000-fold with the blocking buffer and added 50 µL to each well and incubated for two hours at 37 °C. Plates were then washed with 0.2 mL per well 0.1% Tween 20/PBS five times and followed by adding 50 µL per well of horseradish peroxidase (HRP) conjugated goat anti-mouse IgG antibody (Invitrogen, Waltham, MA) at 1:4000 dilution in the blocking buffer. After 1-h incubation at 37 °C, plates were washed five times with 0.2 mL per well 0.1% Tween-20 in PBS. The HRP signals were developed by adding 0.1 mL per well of 3,3',5,5'-tetramethylbenzidine (TMB) reagent (Thermo Fisher Scientific) and incubating at room temperature until the positive control was apparent. At that point, the reaction was stopped by adding 0.1 mL/well TMB stop solution (LGC SeraCare, Milford, MA). The signals were measured by absorbance at 450 nm with an EnVision plate reader (Perkin Elmer, Waltham, MA). The data were analysed using Prism software (GraphPad Software Inc., San Diego, CA).

To test compound cytotoxicity, Huh-7 cells were plated in 96-well plates (Corning Life Sciences) at 0.1 mL/well at 0.144 × 10^6^ cells/mL in growth medium. After the cells were incubated at 37 °C in a humid atmosphere containing 5% (v/v) CO_2_ overnight, 0.1 mL per well growth medium was added to each well along with serial diluted test compounds dispensed by a HP D300e digital dispenser (Hewlett Packard) with a final volume of 0.2 mL/well. The culture was maintained in a humidified chamber at 33 °C with 5% (v/v) CO_2_ for 72 h. After the incubation, Huh-7 cell viability was measured using the CellTiter-Glo Luminescent Cell Viability Assay kit (Promega, Madison, WI) according to the manufacturer’s protocol. The luminescence signals were recorded by an EnVision plate reader. The relative cell viability was calculated by normalizing the absorbance of the compound-treated wells to those of the DMSO-treated wells (set as 100%). The relative cell viability (Y-axis) versus the log10 values of compound concentration (X-axis) were plotted in software Prism (version 8). The CC_50_ (the concentration of test compounds required to reduce cell viability by 50%) values were calculated using a nonlinear regression model (four parameters).

#### Coronavirus 229E and the corresponding cytotoxicity assay

Human coronavirus 229E (HCoV-229E) anti-viral assay was an anti-nucleoprotein ELISA with infected H1 HeLa cells. H1 HeLa cells were seeded into 96-well plates (Corning) at 0.1 mL /well at 0.12 × 10^6^ cells/ml in Opti-MEM (Gibco, Waltham, MA) supplemented with 2% FBS, and were incubated at 37 °C in a humidified tissue culture chamber containing 5% (v/v) CO_2_ overnight. Next day, HCoV-229E stock were diluted to 1.283 × 10^4^ pfu/mL with 2% FBS in Opti-MEM and distributed to the 96-well plates at 0.1 mL per well. Test compounds were distributed to each well using a HP D300e digital dispenser with a final volume of 200 µL/well. After the plates were centrifuged at 700 g for one hour at room temperature, they were maintained in a humidified chamber at 33 °C with 5% (v/v) CO_2_ for 0 or 96 h. After the incubation, the medium was removed, and the plates were fixed with 100% methanol for 10–15 min at room temperature. Then the fixative was removed, and the plates were air dried for 15–30 min. Afterwards, the plates were blocked with 0.1 ml/well 10% FBS (HyClone), 5% dry milk (AmericanBio), 0.1% Tween 20 (EMD Millipore) in PBS (Blocking buffer) for 30 min at 37 °C. After the blocking buffer was removed, 0.05 mL/well 5000 fold diluted HCoV-229E nucleoprotein rabbit polyclonal antibody (Sino Biological, Beijing, China) in the blocking buffer was added to each well and incubated for 2 h at 37 °C. Plates were then washed with 0.2 mL per well 0.1% Tween-20/PBS five times and followed by adding 50 µL per well of HRP conjugated goat anti-rabbit IgG antibody (ImmunoReagents, Raleigh, NC) 1:4000 diluted in the blocking buffer. After one hour incubation at 37 °C, plates were washed five times with 0.2 mL per well 0.1% Tween 20 in PBS. The HRP signals were developed by adding 0.1 mL per well of TMB reagent (Thermo Scientific) and incubating at room until the positive control was apparent. At that point, the reaction was stopped by adding 0.1 mL/well TMB stop solution (LGC SeraCare, Milford, MA). The signals were measured with absorbance at 450 nm with an EnVision plate reader. After the background signals at 0 h was subtracted from those of at 96 h, the relative absorbance was calculated by normalizing the absorbance of the compound-treated groups to that of the DMSO-treated groups (set as 100%). EC_50_ values were calculated using a nonlinear four parameter variable slope regression model.

To test compound cytotoxicity, H1 HeLa cells were plated in 96-well plates (Corning Life Sciences) at 0.1 mL/well at 0.12 × 10^6^ cells/mL in growth medium. After the cells were incubated at 37 °C in a humid atmosphere containing 5% (v/v) CO_2_ overnight, 0.1 mL per well growth medium was added to each well along with serial diluted test compounds dispensed by a HP D300e digital dispenser (Hewlett Packard) with a final volume of 0.2 mL/well. The culture was maintained in a humidified chamber at 33 °C with 5% (v/v) CO_2_ for 96 h. After the incubation, H1 HeLa cell viability was measured using the CellTiter-Glo Luminescent Cell Viability Assay kit (Promega) according to the manufacturer’s protocol. The luminescence signals were recorded by an EnVision plate reader. The relative cell viability was calculated by normalizing the absorbance of the compound-treated wells to those of the DMSO-treated wells (set as 100%). The relative cell viability (Y-axis) versus the log10 values of compound concentration (X-axis) were plotted in software Prism (version 8). The CC_50_ (the concentration of test compounds required to reduce cell viability by 50%) values were calculated using a nonlinear regression model (four parameters).

### HCV 1b and 2a replicon with luciferase reporter

Anti-hepatitis C virus activity was determined by inhibition of HCV GT1b and HCV GT2a replicons in Huh-7 cell lines with a high-throughput 384 well-based luciferase reporter assay. GT1b and 2a subgenomic replicon cell lines. Both cell lines were derived from Huh-7-lunet (Con1/SG-hRlucNeo) stably harboring a HCV genotype 1b or 2a subgenomic replicon with Renilla luciferase as a reporter^41^.

The cell lines were maintained in DMEM supplemented with GlutaMAX (Invitrogen, Carlsbad, CA), 10% FBS (Hyclone Laboratories, not heat-inactivated), 0.5 mg/mL Geneticin (G418) (Invitrogen, Carlsbad, CA), 100 Units/mL penicillin 100 µg/mL streptomycin (Invitrogen, Carlsbad, CA), and 0.1 mM NEAA (Invitrogen, Carlsbad, CA). In the assay, 2000 cells/well in 90 µL cell culture medium without G418 were plated in 384-well assay plates (Greiner Bio-One, Monroe, NC; cell-culture treated). Compounds were serially diluted (1:3) in DMSO and 0.4 µL of diluted compound was added to each well, generating a 10-point dose titration that ranges from 2.3 nM to 44 µM and a final DMSO concentration of 0.44%. Compounds were tested in quadruplicate wells. DMSO vehicle was used as a negative (solvent; no inhibition) control, and a combination of three HCV inhibitors, including a protease inhibitor, an NS5A inhibitor, and a nucleoside inhibitor, was used at concentrations > 100 × EC_50_ as a positive control (100% inhibition). Plates were incubated for 3 days at 37 °C in an atmosphere of 5% CO_2_ and 85% humidity.

The HCV replicon assay was a multiplex assay which assessed cytotoxicity (CC_50_) in addition to anti-replicon activity (EC_50_) in the same well. The anti-HCV replication activity was determined by the luminescence signal generated from the reporter Renilla luciferase of the HCV replicon. The cytotoxicity effect was determined by calcein AM conversion to fluorescent product. Normalized activities in the assays were calculated using Eq. (1):

1 $$y = 100\times \left(1-\frac{{X}_{C}-{M}_{B}}{{M}_{D}-{M}_{B}}\right),$$

where y = normalized % cytotocity or HCV replicon inhibition, *X*_*C*_ = fluorescence signal from compound treated well; *M*_*B*_ = fluorescence signal in positive control wells (100% inhibition), *M*_*D*_ = fluorescence signal in DMSO treated wells (0% inhibition).

To determine corresponding CC_50_ and EC_50_ values, dose responses were analyzed by 4-parameter non-linear regression curve fitting using Eq. (2):

2 $$y=\frac{100 \times {IC}_{50}^{ m}}{{IC}_{50}^{ m}+ {[I]}^{m}},$$

where y = % cyototoxicity or replicon inhibition, m = Hill coefficient, [I] = inhibitor concentration, IC_50_ = CC_50_ for cytotoxicity and EC_50_ for HCV replicon inhibition, respectively.

#### Antiviral activity against DENV-1 (Western Pacific), DENV-2 (New Guinea C), DENV-3 (VN32), DENV-4 (MY01), YFV (YFS11), ZIKV (PRVABC59 and Dakar), and JEV carrying NanoLuc luciferase reporters

These assays were conducted following published methods^42^. Huh-7 cells were seeded at 1.5 × 10^4^ cells per well with 2% FBS in 96-well plate. After incubation overnight, cells were infected with NanoLuc reporter viruses in the presence of twofold serial dilutions of RDV. At 48 h post infection, cells were washed three times with PBS, followed by addition of NanoGlo substrate diluted 1:100 in NanoGlo Assay buffer. After 3 min of incubation at room temperature, plates were read by a BioTek Cytation 5 plate reader. All NanoLuc reporter virus infection assays were performed in a biosafety level 2 (BSL-2) laboratory.

Huh-7 cell viability was measured using the CellTiter-Glo^®^ Luminescent Cell Viability Assay kit (Promega, Madison, WI) according to the manufacturer’s protocol. Huh-7 cells were seeded at 1.5 × 10^4^ cells per well with 2% FBS in a 96-well plate. After overnight incubation at 37 °C in 5% CO_2_, the cells were treated with twofold serial dilutions of the compound. At 48 h post treatment, CellTiter-Glo reagents were added into each well and luminescence signal were recorded by a BioTek Cytation 5 plate reader. The relative cell viability was calculated by normalizing the absorbance of the compound-treated wells to those of the DMSO-treated wells (set as 100%). The relative cell viability (Y-axis) versus the log_10_ values of compound concentration (X-axis) were plotted in software Prism (version 8). The CC_50_ (the concentration of test compounds required to reduce cell viability by 50%) values were calculated using a nonlinear regression model (four parameters).

#### Antiviral activity against DENV-1 Djibouti, DENV-2 RL, DENV-3 H87, DENV-4 Dakar, YFV 17D, and ZIKV MR766

Virus yield reduction assays for DENV-1 Djibouti D1/H/IMTSSA/98/606 (GenBank Accession AF298808)^43^, DENV-2 RL (GenBank Accession MW741553), DENV-3 H87 prototype strain (GenBank Accession M93130)^44^, DENV-4 Dakar_HD_34460 (GenBank Accession KF907503), and ZIKV MR766 (GenBank Accession DQ859059) using Huh-7 cells were performed as follows: Huh-7 cells were seeded at a density of 5.5 × 10^3^ (DENV-1, DENV-2, DENV-3, DENV-4) or 1.5 × 10^3^ (ZIKV) cells per well in 100 μL DMEM/10% FBS culture medium in 96-well plates. The next day, cells were infected at an MOI of 0.01 with DENV-1, DENV-2, DENV-3, DENV-4 or ZIKV diluted in DMEM/5% FBS assay medium (150 μL per well). Cells were incubated for 2 h, after which the viral inoculum was removed. After rinsing the cells with assay medium, twofold serial dilutions (concentration ranged from 100 to 0.078 μM) of RDV in DMEM/5% FBS assay medium were added to the cells. After an incubation period of 4 (DENV-1, DENV-2, DENV-3, and DENV-4) or 7 (ZIKV) days, the supernatant was collected, and the viral RNA load was determined by quantitative RT-PCR (RT–qPCR) as previously described^45,46^. The antiviral activity of RDV against YFV-17D Stamaril (lot G5400; Sanofi-Pasteur) was determined in a cytopathic effect (CPE)-reduction assay. The assay was performed as for DENV-1 to DENV-4, except that RDV was tested at concentrations ranging from 100 to 0.039 μM, and readout (on day 4 post-infection) was done by colorimetric readout using the MTS/PMS method (Promega), as previously described^47^. The EC_50_, which is defined as the compound concentration that is required to inhibit viral RNA replication (for DENV and ZIKV) or the virus-induced CPE (for YFV) by 50%, was determined using logarithmic interpolation.

### Wild-type WNV (New York 99 strain)-based viral titer reduction assay

All experiments were performed in a BSL-3 laboratory. Huh-7 cells were infected with wild-type WNV at an MOI of 0.1. The infected cells were treated with different concentrations of RDV. At 48 h post infection, culture fluids were collected. The viral titer was determined by plaque assay on Vero cells^48^. Huh-7 cell viability was measured as described in Section “Antiviral activity against DENV-1 (Western Pacific), DENV-2 (New Guinea C), DENV-3 (VN32), DENV-4 (MY01), YFV (YFS11), ZIKV (PRVABC59 and Dakar), and JEV carrying NanoLuc luciferase reporters” .

### Antiviral activity against enteroviruses, rhinoviruses, and influenza viruses

Antiviral assessments of RDV for enteroviruses, rhinoviruses, and influenza viruses were conducted in RD, H1 HeLa, and MDCK cells, respectively, using cytopathic effect (CPE) assays. Virus and cells were mixed in the presence of test compound and incubated for the indicated duration. The virus was pre-titered such that control wells exhibited 85 to 95% loss of cell viability due to virus replication. Therefore, antiviral effect or cytoprotection was observed when compounds prevented virus replication. Cytoprotection and compound cytotoxicity was assessed by MTS (CellTiter®96 Reagent, Promega) dye reduction. The % reduction in viral cytopathic effects (CPE) was determined and used to calculate EC_50_ (concentration inhibiting virus replication by 50%) and CC_50_ (concentration resulting in 50% cell death).

### Antiviral activity against filoviruses

The cell-based antiviral assays were conducted in 384- or 96-well plates in BSL-4 containment using a high content imaging system to quantify virus antigen production as a measure of virus infection. “No virus” control wells (BC) and “0.5% DMSO” control wells (NC) were included on each plate to determine the 0 and 100% virus infection signal, respectively.

For the filoviruses assays, ten serial dilutions of compound in triplicate were added directly to the cells using the HP D300 digital dispenser in twofold serial dilution increments starting at 10 μM at 2 h prior to infection. Alternatively, a twofold serial dilution series with 8 concentrations of RDV from 2.5 µM to 20 nM was used. The DMSO concentration in each well was normalized to 0.5% using an HP D300 digital dispenser. The assay plates were transferred to the BSL-4 suite and infected with SUDV and MARV isolates at an MOI which resulted in 20% to 90% of the cells expressing virus antigen at the assay endpoint (Table S4). Infection was terminated by fixing the cells in formalin solution 48 or 72 h after virus inoculation and prior to immunostaining.

The primary and secondary antibodies and dyes used for nuclear and cytoplasmic staining are listed in Table S5. The primary antibody specific for a particular viral antigen was diluted 1000-fold in blocking buffer (1 × phosphate-buffered saline [PBS; VWR, Bridgeport, NJ] with 3% bovine serum albumin [Lampire Biological Laboratories, Pipersville, PA]) and added to each well of the assay plate. The assay plates were incubated for 60 min at room temperature. The primary antibody was removed and the cells were washed 3 times with 1 × PBS. Cells were then incubated with the secondary detection antibody, an anti-mouse immunoglobulin G conjugated with Dylight488 (Thermo Fisher Scientific) at 1 to 1000 dilution in blocking buffer. The assay plates were incubated for 60 min at room temperature. Nuclei and cytoplasm were stained with Hoechst 33,342 (Thermo Fisher Scientific) and HCS CellMask Deep Red (Thermo Fisher Scientific) for nuclei and cytoplasm detection, respectively. At least five images per well were acquired by an Opera confocal plate imaging instrument (Perkin Elmer, Waltham, MA) using a 10 × air objective. Signals from virus antigen, nuclei, and cytoplasm staining were detected at 488-, 400-, or 640-nm emission wavelengths, respectively. 

Image analysis was performed using Acapella imaging software (Perkin Elmer, Waltham, MA), provided within the Opera imaging system (model 3842, quadruple excitation high sensitivity [QEHS]; Perkin Elmer). The software was used to capture well-based output parameters that were used to calculate the percent of infected cells, percent inhibition and percent viability. The percent of infected cells was calculated using Eq. (3) below

3 $$\% Virus\, positive = \frac{Number\, of\, Virus \,Positive\, Cells (S)}{Number\, of\, Nuclei\, (S)}*100.$$

Number of Virus Positive Cells (S) refers to the number of cells in a sample (S) well with virus-specific signal above the threshold value, while Number of Nuclei (S) refers to the number of cells in a sample well (S) with nuclear staining signal above the threshold value. The % Virus positive cells is calculated by Acapella directly which indicates the median percentage of cells in a sample (S) well with virus-specific fluorescence intensity signal above the threshold value.

Dose–response curve analysis was performed using Genedata Screener software (version 18.0, Lexington, MA, https://www.genedata.com/products-services/screener) applying the Levenberg–Marquardt algorithm for curve-fitting strategy. Most of the curve fittings were done using 2-, 3- or 4-parameter nonlinear regression. The following values were calculated for each curve: absolute EC_50_ value, equal to 50% inhibition of infection; standard deviation for EC_50_ within 3 or more technical replicates; Hill slope, used for calculation of EC_90_ values; %CC giving CC_50_ values that are equal to 50% of cell counts; and selectivity index (SI), representing the ratio of CC_50_ to EC_50_.

### Antiviral activity against hepatitis B virus and corresponding cytotoxicity in HepG2 and primary human hepatocytes (PHH)

The HepG2-NTCP cells were generated by transfection of plasmid DNA encoding human sodium taurocholate cotransporting polypeptide (NTCP) gene into HepG2 cells. Clone HY12 was selected with G418 and expanded in cell culture for HBV infection. Cells were infected by a genotype D HBV virus derived from HepAD38 cells (AD38 virus) at 5000 genome equivalents per cell in the presence of 4% PEG 8000 and 1.5% DMSO for 20 h and incubated in the medium with 1.5% DMSO for 3 days after virus inoculums were removed^49^. Infected cells were plated into 384-well plates (20,000 cells/well), treated with compounds for 5 days, and followed with measurement of the HBsAg in the supernatant using Meso Scale Diagnostics (MSD) (Rockville, MD) detection kits as described previously^50^.

The anti-HBV activity assay in PHH was performed in a 1536-well format using a published method^50^. PHH cells were infected by AD38 virus at 500 genome equivalents per cell for 3 days and then trypsinized and plated into 1536-well plates (Greiner Bio-One, Monroe, NC; cell-culture treated) at 4000 cells/well with collagen coating (Gibco/Thermo Fisher Sciences) of 1 μL at 25 μg/mL before cell seeding. Compounds are serially diluted (1:3) in DMSO. 10 nL of diluted compound is added to each well, starting at a final concentration of 1 μM with a final DMSO concentration of 1.5% in a total volume of 9 μL culture medium. For each drug concentration, quadruple wells were set up in the 1536-well plate. Infected PHH cell with 1.5% DMSO was used as a negative control, and un-infected PHH cells was used as a positive control (100% inhibition). Plates were incubated for 3 days at 37 °C in an atmosphere of 5% CO_2_ and 85% humidity.

6 mL of cell supernatants were transferred and half of the supernatants were utilized to measure HBsAg in another 1536-well plate (Corning) by incubating with 5 mL of detection HBs surface antigen antibody mix of customer tagged with Tb and D2 and detecting time-resolved fluoresce on Envision plate reader (PerkinElmer). Cell toxicity was detected by adding 1 mL of 2´ CellTiter-Glo (Promega) solution into each well of the cell plate.

The EC_50_ values were determined as the testing compound concentration that caused a 50% decrease in HTRF signal. The CC_50_ values were determined as the testing compound concentration that caused a 50% decrease of cell viability.

### Antiviral activity against hepatitis delta virus and corresponding cytotoxicity in Huh7-NTCP cells

The Huh7-NTCP cells were generated by transfection of plasmid DNA encoding human sodium taurocholate cotransporting polypeptide (NTCP) gene into Huh7 cells. A single cell clone with stable NTCP expression when cultured in the presence of G418 was selected. IF detection of HDV replication was performed using the procedure previously described by Ni et al.^51^ with modifications. Briefly, the cells were bulk infected at 37 C for 16 h with HDV genotype 1 virus produced from Huh7-END cells^52^ (kindly provided by Stephen Urban, University Hospital Heidelberg, Germany) at a MOI = 1 in high glucose DMEM (Gibco) media containing 4% PEG 8000, 2.5% DMSO, 10% FBS, 100 unit/mL penicillin, 100 units/mL streptomycin, and 2 mM L-glutamine. The cells were then trypsinized and plated at a density of 2000 cells/well into 1536 well plates (Corning) containing DMSO solutions of the compound at varying concentrations in DMEM supplemented with 10% FBS, 2.5% DMSO, 100 units/mL penicillin, and 100 units/mL streptomycin for 4 days. Following treatment, cells were fixed with 5% formaldehyde and stained for HDAg using mouse anti-HDAg antibody (kindly provided by Stephen Urban, University Hospital Heidelberg, Germany) and AlexaFluor647 conjugated goat anti-mouse secondary antibody (Invitrogen). Cells were counterstained with Hoechst 33,342 (Invitrogen) to identify nuclei.

The cells were imaged using ThermoFisher CellInsight CX7 High Content Analysis platform with a 10 × objective. HDAg was read at 650 nm and the average intensity of the nuclear regions was used to identify HDV infected cells. The fraction of cells with average intensity above the threshold set at approximately 5 standard deviations above the value mean value for the uninfected control was reported for each condition and used to monitor inhibition of HDV.

Nuclei count was read at 405 nm and used as a marker for cytotoxicity.

GraphPad Prism 8 software was used for the data analysis. The results were normalized to control wells containing DMSO only. A nonlinear regression was used to fit a 4-parameter (variable slope) model to the data.

### Antiviral activity against hepatitis E replicon and corresponding cytotoxicity evaluation

Huh-7 cells are grown in DMEM (Gibco/Thermo Fisher Sciences) supplemented with 10% FBS (Hyclone Laboratories) in a humidified 5% CO_2_ incubator at 37 °C. When Huh-7 cells are 90–95% confluent, single-cell suspensions of Huh-7 cells are prepared by trypsinization, washed twice with serum-free Opti-MEM^®^ (Invitrogen) and resuspended at 4 × 10^6^ cells in 200 μL BTXpress™ buffer (VWR, Leuven, Belgium).

HEV Kernow-C1 p6/luc-encoding RNA is in vitro transcribed from MluI-digested plasmid DNA using the T7 RiboMAX Large Scale RNA production system (Promega) and subsequently capped using the ScriptCap m7G capping system (Cellscript, Madison, WI).

To determine RDV antiviral activity, 5 μg of capped Kernow-C1 p6/Luc RNA is carefully mixed with the aforementioned cell suspension in a 4 mm cuvette (VWR International). An ECM 830 Electro Square Porator™ (BTX Harvard Apparatus, Holliston, MA) is used to deliver 5 pulses at 900 V, 99 μsec. For cell control wells, viral RNA is omitted. Cells are immediately transferred to 20 mL complete DMEM and seeded as required for the assay. Briefly, 100 μL aliquots of the cell suspension are seeded in 96-well plates already containing serial dilutions of RDV (100 μL, 2 × concentrated) in complete DMEM. After 4 days culture at 37 °C, the level of viral replication was determined by the luciferase signal in the cell culture supernatant. Twenty μL of the culture medium is transferred to a white 96-well culture plate (PerkinElmer) and luminescence produced by the secreted Gaussia luciferase (*Gluc*) is determined after addition of 50 μL Renilla luciferase assay substrate (1:100 diluted; Promega). The 50% effective concentration (EC_50_) is defined as the concentration of compound that causes a 50% reduction in luminescence (*Luc* signal) compared to that of the virus controls using [Inhibitor] vs. normalized response—Variable slope curve fitting (GraphPad Prism, version 8).

For toxicity evaluation, medium is removed and replaced with 100 μL of a 3-(4,5- dimethylthiazol-2-yl)-5-(3-carboxymethoxyphenyl)-2-(4-sulfophenyl)-2-tetrazolium/phenazinemethosulfate (MTS/PMS; Promega) solution (1:10 diluted). Metabolically active cells convert the yellow substrate into a brown, water-soluble metabolite. The amount of metabolite that is produced, directly correlates to the metabolic activity of the cells either in the presence of compound or in the presence of both compound and virus. After an incubation period of 1 h at 37 °C, the optical density at 498 nm is determined for each well. The MTS readout is followed by microscopic evaluation (quality control). The CC_50_ represents the concentration at which the metabolic activity of the cells would be reduced to 50% of the metabolic activity of untreated cells, using [Inhibitor] vs. normalized response—Variable slope curve fitting (Graphpad Prism, version 8).

### Antiviral effects of combination remdesivir + favipiravir for SUDV and MARV

HeLa cells, 384-well plates, and viral stocks (MARV Ci67 and SUDV Boniface) are the same as described above. Favipiravir was obtained from Fujifilm. Compounds were added by HP D300 (Hewlett Packard) digital dispenser from a 100% dimethyl sulfoxide (DMSO) stock so that each combination was tested in 4 replicates. Favipiravir was added 4 h after cell plating (18–20 h prior to virus inoculation) at six concentrations ranging from 23.4 nM to 750 µM. RDV was added to cells 2 h before virus inoculation at 9 different concentrations ranging from 4 nM to 1 µM. E864-0360, a USAMRIID control compound with EC_50_ of 0.40 and 0.65 µM against MARV, RAVV, and SUDV Boniface, respectively, was added 2 h before virus inoculation at 10 difference concentrations ranging from 50 nM to 25 µM. A no-virus inoculation blank control, a neutral control with virus infection but no treatment, and a vehicle control with virus infection and DMSO only were included for establishing reference values for analysis. Cells were fixed in 10% buffered formalin at least 48 h before immunostaining. HCI measure of virus infection was described above.

The combinatorial effect (additive, synergistic, or antagonistic) of favipiravir and RDV was evaluated using a conventional checkerboard set up, where one compound’s dose–response was evaluated in the presence of increasing concentrations of the other compound. Dose–response of RDV in the absence of favipiravir and vice versa was performed in the same assay to provide control for evaluation of the combination effect. Genedata Screener Synergy Module analytic software (Basel, Switzerland) was used to quantify the effect of combination using HSA (Highest Single Agent), Bliss independence, and Loewe mathematic models^53^. To minimize software bias, we also analyzed the combination of RDV + favipiravir for anti-MARV activity using SynergyFinder (version 3.0)^30^ where synergy scores are reported. Based on the conventional cut-off, a synergy score of -10 to 10 are considered additive.

Three parameters were used to evaluate the combination effect depending on the different models: (1) Combination index (CI) measures the fractional shift between the combination doses and single agents’ percentage activity. It is independent of a model for the joint action of the two compounds. A CI value of 1 indicates additive effect, a value less than 1 indicates synergistic effects, and a value greater than 1 indicates antagonism between the two compounds; (2) Excess volume refers to the sum of the differences between the model and the fitted data, multiplied by the concentration differences between the single measurements. Positive values point toward synergistic effects, a value near zero indicates a neutral combination effect, and negative values point toward antagonism; (3) Synergy score is similar to the excess volume, except that it weights the data by activity values from the original measured data, where positive values indicate relative levels of synergy, and negative values indicate relative levels of antagonism.

### Remdesivir interactions with concomitant medications

Therapeutic agents used in SUDV- and MARV-endemic regions were evaluated for potential interactions with RDV (Table S3). The drugs include HIV antiviral therapies, antimalarial drugs, and medications used to ameliorate symptom of viral hemorrhagic fevers (WHO 2010, 2016). HeLa cells, 384-well plates, and viral stocks (MARV Ci67 and SUDV Boniface) are the same as described above. Each compound was tested at 8 concentrations in 3–4 replicates with a threefold step dilution. Each concentration of each compound was added to assay wells from a stock solution using an HP D300 (Hewlett Packard) digital dispenser. A no-virus inoculation blank control, a neutral control with virus infection but no treatment, a vehicle control with virus infection, DMSO only, and positive control E864-0360 were included for establishing reference values for analysis. Two dose–response studies were performed for each compound.

Two hours after treatment, the plates were transferred to a BSL-4 laboratory for inoculations with SUDV Boniface (MOI = 7–16 pfu/mL) or MARV Ci67 (MOI = 5–9 pfu/mL) and incubated for 48 h. Infection was terminated by formalin fixation. Infections was measured by HCI as described above. Analysis was performed as described above in this Section “Remdesivir interactions with concomitant medications”.

## Supplementary Information

Supplementary Information.

## Data Availability

The datasets generated during and/or analyzed during the current study are available from the corresponding author on reasonable request.

## References

[1] Sheahan TP (2017). Broad-spectrum antiviral GS-5734 inhibits both epidemic and zoonotic coronaviruses. Sci. Transl. Med..

[2] Agostini ML (2018). Coronavirus susceptibility to the antiviral Remdesivir (GS-5734) is mediated by the viral polymerase and the proofreading exoribonuclease. mBio.

[3] Pruijssers AJ (2020). Remdesivir inhibits SARS-CoV-2 in human lung cells and chimeric SARS-CoV expressing the SARS-CoV-2 RNA polymerase in mice. Cell Rep..

[4] Sheahan TP (2020). Comparative therapeutic efficacy of remdesivir and combination lopinavir, ritonavir, and interferon beta against MERS-CoV. Nat. Commun..

[5] Sheahan TP (2020). An orally bioavailable broad-spectrum antiviral inhibits SARS-CoV-2 in human airway epithelial cell cultures and multiple coronaviruses in mice. Sci. Transl. Med..

[6] Xie X (2020). A nanoluciferase SARS-CoV-2 for rapid neutralization testing and screening of anti-infective drugs for COVID-19. Nat. Commun..

[7] Martinez DR (2021). Prevention and therapy of SARS-CoV-2 and the B.1.351 variant in mice. Cell Rep..

[8] Ye W (2020). Remdesivir (GS-5734) impedes enterovirus replication through viral RNA synthesis inhibition. Front. Microbiol..

[9] Porter DP (2020). Remdesivir (GS-5734) is efficacious in cynomolgus macaques infected with Marburg virus. J. Infect. Dis..

[10] Warren TK (2016). Therapeutic efficacy of the small molecule GS-5734 against Ebola virus in rhesus monkeys. Nature.

[11] Siegel D (2017). Discovery and synthesis of a phosphoramidate prodrug of a Pyrrolo [2,1-f][triazin-4-amino] Adenine C-Nucleoside (GS-5734) for the treatment of ebola and emerging viruses. J. Med. Chem..

[12] Mackman RL (2021). Prodrugs of a 1'-CN-4-Aza-7,9-dideazaadenosine C-Nucleoside leading to the discovery of Remdesivir (GS-5734) as a potent inhibitor of respiratory syncytial virus with efficacy in the African green monkey model of RSV. J. Med. Chem..

[13] Lo MK (2017). GS-5734 and its parent nucleoside analog inhibit Filo-, Pneumo-, and Paramyxoviruses. Sci. Rep..

[14] Lo MK (2019). Remdesivir (GS-5734) protects African green monkeys from Nipah virus challenge. Sci. Transl. Med..

[15] Gordon CJ (2022). Efficient incorporation and template-dependent polymerase inhibition are major determinants for the broad-spectrum antiviral activity of remdesivir. J. Biol. Chem..

[16] Brown AJ (2019). Broad spectrum antiviral remdesivir inhibits human endemic and zoonotic deltacoronaviruses with a highly divergent RNA dependent RNA polymerase. Antivir. Res..

[17] Hsu HY (2021). Remdesivir and cyclosporine synergistically inhibit the human coronaviruses OC43 and SARS-CoV-2. Front. Pharmacol..

[18] Hamre D, Procknow JJ (1966). A new virus isolated from the human respiratory tract. Proc. Soc. Exp. Biol. Med..

[19] Cho A (2012). Synthesis and antiviral activity of a series of 1'-substituted 4-aza-7,9-dideazaadenosine C-nucleosides. Bioorg. Med. Chem. Lett..

[20] Warren TK (2021). Remdesivir is efficacious in rhesus monkeys exposed to aerosolized Ebola virus. Sci. Rep..

[21] Beitzel BF (2021). On-demand patient-specific phenotype-to-genotype Ebola virus characterization. Viruses.

[22] Pessi A (2019). Cholesterol-conjugated stapled peptides inhibit Ebola and Marburg viruses in vitro and in vivo. Antivir. Res..

[23] Furuta Y (2009). T-705 (favipiravir) and related compounds: Novel broad-spectrum inhibitors of RNA viral infections. Antivir. Res..

[24] Oestereich L (2014). Evaluation of antiviral efficacy of ribavirin, arbidol, and T-705 (favipiravir) in a mouse model for Crimean-Congo hemorrhagic fever. PLoS Negl. Trop. Dis..

[25] Ison MG (2015). Optimizing antiviral therapy for influenza: Understanding the evidence. Expert Rev. Anti. Infect. Ther..

[26] Yen HL (2016). Current and novel antiviral strategies for influenza infection. Curr. Opin. Virol..

[27] Madelain V (2016). Ebola virus infection: Review of the pharmacokinetic and pharmacodynamic properties of drugs considered for testing in human efficacy trials. Clin. Pharmacokinet..

[28] Du YX, Chen XP (2020). Favipiravir: Pharmacokinetics and concerns about clinical trials for 2019-nCoV infection. Clin. Pharmacol. Ther..

[29] Nguyen TH (2017). Favipiravir pharmacokinetics in Ebola-Infected patients of the JIKI trial reveals concentrations lower than targeted. PLoS Negl. Trop. Dis..

[30] Ianevski A, Giri AK, Aittokallio T (2022). SynergyFinder 3.0: An interactive analysis and consensus interpretation of multi-drug synergies across multiple samples. Nucleic Acids Res..

[31] Barrett R, Kuzawa CW, McDade T, Armelagos GJ (1998). Emerging and Re-emerging infectious diseases: The third epidemiologic transition. Annu. Rev. Anthropol..

[32] Satcher D (1995). Emerging infections: Getting ahead of the curve. Emerg. Infect. Dis..

[33] GOV.UK. (2019).

[34] Kleandrova VV, Speck-Planche A (2021). The urgent need for pan-antiviral agents: From multitarget discovery to multiscale design. Future Med. Chem..

[35] Wang B, Svetlov D, Bartikofsky D, Wobus CE, Artsimovitch I (2022). Going retro, going viral: Experiences and lessons in drug discovery from COVID-19. Molecules.

[36] Hickman MR, Saunders DL, Bigger CA, Kane CD, Iversen PL (2022). The development of broad-spectrum antiviral medical countermeasures to treat viral hemorrhagic fevers caused by natural or weaponized virus infections. PLoS Negl. Trop. Dis..

[37] Lamb YN (2020). Remdesivir: First approval. Drugs.

[38] VEKLURY & Gilead Sciences Inc. (2022).

[39] Cox RM (2021). Oral prodrug of remdesivir parent GS-441524 is efficacious against SARS-CoV-2 in ferrets. Nat. Commun..

[40] Schafer A (2022). Therapeutic treatment with an oral prodrug of the remdesivir parental nucleoside is protective against SARS-CoV-2 pathogenesis in mice. Sci. Transl. Med..

[41] Robinson M (2010). Novel HCV reporter replicon cell lines enable efficient antiviral screening against genotype 1a. Antimicrob. Agents Chemother..

[42] Baker C (2020). Identifying optimal capsid duplication length for the stability of reporter flaviviruses. Emerg. Microbes Infect..

[43] Tolou HJG (2001). Evidence for recombination in natural populations of dengue virus type 1 based on the analysis of complete genome sequences. J. Gen. Virol..

[44] Osatomi K, Sumiyoshi H (1990). Complete nucleotide sequence of dengue type 3 virus genome RNA. Virology.

[45] De Burghgraeve T (2013). 3',5'Di-O-trityluridine inhibits in vitro flavivirus replication. Antivir. Res..

[46] Zmurko J (2016). The viral polymerase inhibitor 7-Deaza-2'-C-Methyladenosine is a potent inhibitor of in vitro Zika virus replication and delays disease progression in a robust mouse infection model. PLoS Negl. Trop. Dis..

[47] Kaptein SJ (2010). A derivate of the antibiotic doxorubicin is a selective inhibitor of dengue and yellow fever virus replication in vitro. Antimicrob. Agents Chemother..

[48] Shan C (2016). An infectious cDNA Clone of Zika virus to study viral virulence, mosquito transmission, and antiviral inhibitors. Cell Host Microbe.

[49] Ni Y (2014). Hepatitis B and D viruses exploit sodium taurocholate co-transporting polypeptide for species-specific entry into hepatocytes. Gastroenterology.

[50] Niu C (2017). The Smc5/6 complex restricts HBV when localized to ND10 without inducing an innate immune response and is counteracted by the HBV X protein shortly after infection. PLoS ONE.

[51] Ni Y (2019). Generation and characterization of a stable cell line persistently replicating and secreting the human hepatitis delta virus. Sci. Rep..

[52] Lempp FA (2019). Recapitulation of HDV infection in a fully permissive hepatoma cell line allows efficient drug evaluation. Nat. Commun..

[53] Berenbaum MC (1989). What is synergy?. Pharmacol. Rev..

