# Supplementary material for: Expanded profiling of Remdesivir as a broad-spectrum antiviral and low potential for interaction with other medications in vitro
Source: Sci Rep. 2023 Feb 23;13:3131. doi: 10.1038/s41598-023-29517-9 (PMC9950143; doi:10.1038/s41598-023-29517-9)

**Supplementary Material**

**Expanded Profiling of Remdesivir as a Broad-spectrum Antiviral and Low Potential for Interaction with Other Medications *In Vitro***

Sheli R. Radoshitzky, Patrick Iversen, Xianghan Lu, Jing Zou, Suzanne J.F. Kaptein, Kelly S. Stuthman, Sean A. Van Tongeren, Jesse Steffens, Ruoyu Gong, Hoa Truong, Annapurna A. Sapre, Huiling Yang, Xiaodong Xie, Jia Jun Chia, Zhijuan J. Song, Stacey M. Leventhal, Josolyn Chan, Alex Shornikov, Xin Zhang^4^, David Cowfer, Helen Yu, Travis Warren, Tomas Cihlar, Danielle P. Porter, Johan Neyts, Pei-Yong Shi, Jay Wells, John P. Bilello, and Joy Y. Feng

Table S1: Combination of RDV+favipiravir against SUDV in cell-based antiviral and cytotoxicity evaluation.

| **Dose–Response of RDV across Concentrations of Favipiravir** | | | | | **Dose–Response of Favipiravir across Concentrations of RDV** | | | | |
| --- | --- | --- | --- | --- | --- | --- | --- | --- | --- |
| **Favipiravir Concentration (µM)** | **RDV EC_50_ (µM)** | **SD** | **RDV CC_50_ (µM)** | **RDV SI** | **GS-5734 Concentration (µM)** | **Favipiravir EC_50_ (µM)** | **SD** | **Favipiravir CC_50_ (µM)** | **Favipiravir SI** |
| 0 | 0.209 | 0.033 | >1 | >4.805 | 0 | 507.4 | 79.85 | >750 | >1.372 |
| 23.44 | 0.243 | 0.047 | >1 | >4.117 | 0.004 | 563.2 | 84.13 | >750 | >1.321 |
| 46.88 | 0.212 | 0.041 | >1 | >4.160 | 0.008 | 558.5 | 102.50 | >750 | >1.298 |
| 93.75 | 0.212 | 0.038 | >1 | >3.920 | 0.016 | 546.7 | 72.35 | >750 | >1.224 |
| 187.5 | 0.151 | 0.030 | >1 | >4.664 | 0.031 | 494.6 | 88.27 | >750 | >1.346 |
| 375 | 0.092 | 0.034 | >1 | >6.305 | 0.063 | 402.6 | 96.84 | >750 | >1.379 |
| 750 | <0.004 | — | >1 | >250 | 0.125 | 267.4 | 103.01 | >750 | >1.433 |
|  |  |  |  |  | 0.25 | <23.44 | — | >750 | >32.002 |
|  |  |  |  |  | 0.5 | <23.44 | — | >750 | >32.002 |
|  |  |  |  |  | 1 | <23.44 | — | >750 | >32.002 |

CC_50_, 50% cytotoxic concentration; EC_50_, half maximum effective concentration; FBS, fetal bovine serum; SD, standard deviation; SI, selectivity index.

Table S2: Combination of RDV+favipiravir against MARV in cell-based antiviral and cytotoxicity evaluation.

| **Dose–Response of RDV across Concentrations of Favipiravir** | | | | | **Dose–Response of Favipiravir across Concentrations of RDV** | | | | |
| --- | --- | --- | --- | --- | --- | --- | --- | --- | --- |
| **Favipiravir Concentration (µM)** | **RDV EC_50_ (µM)** | **SD** | **RDV CC_50_ (µM)** | **RDVSI** | **RDV Concentration (µM)** | **Favipiravir EC_50_ (µM)** | **SD** | **Favipiravir CC_50_ (µM)** | **Favipiravir SI** |
| 0 | 0.040 | 0.003 | >1 | >24.254 | 0 | 113.00 | 9.796 | >750 | >6.068 |
| 23.44 | 0.038 | 0.005 | >1 | >23.202 | 0.004 | 104.50 | 15.383 | >750 | >6.591 |
| 46.88 | 0.028 | 0.003 | >1 | >28.433 | 0.008 | 97.88 | 14.367 | >750 | >6.932 |
| 93.75 | 0.013 | 0.004 | >1 | >30.534 | 0.016 | 80.39 | 15.066 | >750 | >7.218 |
| 187.5 | <0.004 | — | >1 | >250 | 0.031 | 44.47 | 16.474 | >750 | >8.361 |
| 375 | <0.004 | — | >1 | >250 | 0.063 | <23.44 | — | >750 | >32.002 |
| 750 | <0.004 | — | >1 | >250 | 0.125 | <23.44 | — | >750 | >32.002 |
|  |  |  |  |  | 0.25 | <23.44 | — | >750 | >32.002 |
|  |  |  |  |  | 0.5 | <23.44 | — | >750 | >32.002 |
|  |  |  |  |  | 1 | <23.44 | — | >750 | >32.002 |

CC_50_, 50% cytotoxic concentration; EC_50_, half maximum effective concentration; FBS, fetal bovine serum; SD, standard deviation; SI, selectivity index.

Table S3: Concomitant medications tested for potential interactions with remdesivir.

| **Compound** | **CAS #** | **Vendor, Catalog #** | **Cmax (ng/mL) [citation]** | **M_W_ (g/mol)** | **Proposed Test Dose (Cmax/M_W_)**  **(µM)** | **Plasma Protein Binding*** | **Treatment Recommendations** |
| --- | --- | --- | --- | --- | --- | --- | --- |
| Acetaminophen  (paracetamol) | 103-90-2 | Tocris, 1706 | 11400 [1] | 151.16 | 75.42 | 10–25% | Common antipyretic, first-line in WHO Viral hemorrhagic fever (VHF) treatment guidelines |
| Amodiaquine hydrochloride dihydrate | 6398-98-7 | Sigma, A2799 | 32.3 [2] | 464.81 | 0.07 | unknown | Used in combination with artesunate as first-line treatment for uncomplicated malaria |
| Artemether | 71963-77-4 | Sigma, A9361 | 198 [3] | 298.37 | 0.66 | 95.4% | Alternative oral treatment for uncomplicated malaria, in combination with lumefantrine |
| Artesunate | 88495-63-0 | Sigma, A3731 | 3260 [4] | 384.42 | 8.48 | 93% | IV antimalarial of choice for severe malaria; used in combination with amodiaquine as first-line treatment for uncomplicated malaria |
| Atovaquone | 95233-18-4 | Sigma, A7986 | 8300 [5, 6] | 366.84 | 22.63 | 99.9% | Most common malaria chemoprophylaxis, in combination with proguanil |
| Ceftriaxone  (ceftriaxone disodium salt hemi(heptahydrate)) | 104376-79-6 | Sigma, C5793 | 291000 [7] | 661.60 | 439.84 | unknown | Recommended common antibiotic in the WHO VHF treatment guidelines; third-generation cephalosporin |
| Ciprofloxacin | 85721-33-1 | Sigma, 17850 | 4600 [8] | 331.34 | 13.88 | 20–40% | Recommended common antibiotic in the WHO VHF treatment guidelines |
| Diazepam | 439-14-5 | Patterson Veterinary,  07-801-4951 | 492 [9] | 284.74 | 1.7 | 98–99% | Commonly used sedative, and as an acute antiepileptic, listed in WHO VHF treatment guidelines |
| Lumefantrine | 82186-77-4 | Sigma, L5420 | 6300 [10] | 528.94 | 1.73 | 99.7% | Alternative oral treatment for uncomplicated malaria, in combination with artemether |
| Metronidazole | 443-48-1 | Sigma, M3761- 5g | 5100 [11] | 171.15 | 11.91 | 20% | Common antibiotic and antiprotozoal medication |
| Omeprazole | 73590-58-6 | Sigma, O104 | 598 [12] | 345.42 | 29.80 | 95% | Common stomach acid suppression medication, listed in WHO VHF treatment guidelines |
| Ondansetron  (ondansetron hydrochloride dihydrate) | 103639-04-9 | Sigma, O3639 | 106 [13] | 365.86 | 0.17 | 73% | Common antinausea medication, in WHO VHF treatment guidelines |
| Proguanil hydrochloride | 637-32-1 | Sigma, G7048 | 918 [5] | 290.19 | 0.29 | unknown | Most common malaria chemoprophylaxis, in combination with atovaquone |
| **Antiretrovirals** | | | | | | | |
| Efavirenz | 154598-52-4 | Gilead Sciences,  GS-017437 | 12900 [14] | 315.67 | 40.86 | 99.5–99.75% | An antiretroviral medication used in combination with other medications to treat and prevent HIV/AIDS |
| Lamivudine  (3TC) | 134678-17-4 | Gilead Sciences,  GS-281429 | 2600 [15] | 229.26 | 11.34 | <36% | An antiretroviral medication used to treat hepatitis B and used in combination with other medications to treat and prevent HIV/AIDS |
| Lopinavir | 192725-17-0 | Gilead Sciences,  GS-016378 | 9800 [16] | 628.81 | 15.58 | >98% | An antiretroviral medication used in combination with ritonavir to treat and prevent HIV/AIDS |
| Ritonavir | 155213-67-5 | Gilead Sciences,  GS-017415 | 1271 [17] | 720.95 | 1.76 | 98–99% | An antiretroviral medication used to treat hepatitis C and used in combination with other medications to treat and prevent HIV/AIDS |
| Tenofovir disoproxil fumarate | 202138-50-9 | Gilead Sciences,  GS-004331 | 300 [18, 19] | 635.52 | 0.47 | <1% | An antiretroviral medication used to treat chronic hepatitis B and used to treat and prevent HIV/AIDS |

Notes: Cmax, maximum serum concentration; IV, intravenous; MARV, Marburg virus; MW, molecular weight; SUDV, Sudan virus; VHF, viral hemorrhagic fever. *Source for plasma protein binding: PubChem compound summaries. Available at: <https://pubchem.ncbi.nlm.nih.gov>. Sources for Cmax: [1] Wattanakul et al. 2016; [2] Winstanley et al. 1987; [3] Chinh et al. 2009; [4] Byakika-Kibwika et al. 2012; [5] Na-Bangchang et al. 2005; [6] Rolan et al. 1994; [7] Acharya et al. 1994; [8] U.S. FDA 2016a; [9] Mandelli et al. 1978; [10] Djimde et al. 2011; [11] Amon et al. 1978; [12] Rhim et al. 2009; [13] U.S. FDA 2016b; [14] U.S. FDA 2005a; [15] U.S. FDA 2017; [16] U.S. FDA 2000; [17] Boffito et al. 2005; [18] Hazra et al. 2004; [19] U.S. FDA 2005b.

Table S4: Multiplicity of Infection (MOI) used in in vitro viral infection assays.

| Virus (strain) | Isolate | GenBank Accession | MOI  (pfu/cell) | Assay endpoint (time post virus exposure) |
| --- | --- | --- | --- | --- |
| OC43 |  |  | 0.04 | 72 h |
| 229E |  |  | 0.05 | 96 h |
| Enterovirus 68D | US/MO/14-18947 |  | U^1^ | 120 h |
| Enterovirus 71 | H |  | U | 96 h |
| Rhinovirus A2 |  |  | U | 96 h |
| Rhinovirus 14 |  |  | U | 96 h |
| Rhinovirus 16 |  |  | U | 96 h |
| DENV-1 (Western Pacific) |  |  | 0.1 | 48 h |
| DENV-1 (Djibouti) | D1/H/IMTSSA/98/606 | AF298808 |  |  |
| DENV-2 (New Guinea C) |  |  | 0.1 | 48 h |
| DENV-2 (RL) |  | MW741553 | 0.01 | 96 h |
| DENV-3 (VN32) |  |  | 0.1 | 48 h |
| DENV-3 (H87) |  | M93130 |  |  |
| DENV-4 (MY01) |  |  |  |  |
| DENV-4 (Dakar_HD_34460) |  | KF907503 | 0.001 | 48 h |
| ZIKV (PRVABC59) |  |  | 0.01 | 48 h |
| ZIKV (Dakar) |  |  | 0.01 | 48 h |
| ZIKV (MR766) |  | DQ859059 | 0.01 | 7 day |
| YFV-Nano |  |  | 0.001 | 48 h |
| YFV-17D Stamaril |  |  | U | 96 h |
| JEV |  |  | 0.001 | 48 h |
| WNV | New York 99 strain |  | 0.1 | 48 h |
| EBOV | Ebola virus/H.sapiens-wt/LBR/2014/Makona-201403261 | KP240932) | 2 | 48 h |
|  | Ebola virus - Mayinga, Zaire, 1976 | AF086833 | 3 |  |
|  | Ebola virus/H. sapiens-tc/COD/1995/Kikwit-9510621 | KU182905 | 2 |  |
| SUDV | Sudan virus/H.sapiens-tc/SDN/2004/Yambio-HCM/SAV/017 | MH121169 | 10 | 48 h |
|  | Sudan virus/H.sapiens-tc/UGA/2000/Gulu-808892 | KR063670 | 7 | 72 h |
|  | Sudan virus/H.sapiens-tc/SDN/1976/Nzara-Boneface | MH121162 | 8 | 48 h |
| MARV | Marburg virus/H.sapiens-tc/GER/1976/Hesse-Cieplik | GQ433353 | 2 | 48 h |
|  | Marburg virus/H.sapiens-tc/AGO/2005/Angola-368) | KY047763 | 2 |  |
|  | Marburg virus/H.sapiens-tc/KEN/1980/Mt. Elgon-Musoke | DQ217792 | 10 |  |
|  | Marburg virus/H.sapiens-tc/UGA/2012/Kabale-Mbg-422-2012 | KC545387 | 2.7 | 72 h |
|  | Marburg virus/H.sapiens-tc/COD/1999/Durba-Nganda 09DRC99 | DQ447652 | 2 | 96 h |
|  | Marburg virus/H.sapiens-tc/COD/1999/Durba-Drandema 05DRC99 | DQ447651 | 0.3 | 72 h |
| RAVV | Ravn virus H.sapiens-tc/KEN/1987/Kitum Cave-810040 | KU179482 | 1.2 |  |
| IAV | A/California/07/2009 |  | U | 72 h |
| IBV | B/Brisbane/60/2008 |  | U | 72 h |
| HBV |  |  |  | 72 h |
| HDV |  |  | 1.0 | 96 h |
| HEV |  |  |  | 96 h |

^1^ U = undefined, the virus was pre-titered such that control wells exhibited 85 to 95% loss of cell viability due to virus replication.

Table S5: List of primary and secondary antibodies used in immune-staining assay

|  | Antibodies for immune staining | | |
| --- | --- | --- | --- |
| Virus | Primary antibody | Secondary antibody | Cell Staining |
| Ebola virus | mm6D8 anti-GP | DyLight488 anti-mouse-IgG | Draq5 (Nuclei) |
| Sudan virus (Bonface, Gulu, and Yambio) | mm3C10 anti-GP |  |  |
| Bundibugyo virus | mmAE11 anti-VP40 |  |  |
| Marburg virus (Ci67) | mm9G4 anti-GP |  |  |
| Marburg virus (Kabale, Nganda, and Drandema), Ravn virus | mm1H11 anti-VP40 |  | Hoechst (Nuclei)  Cell Mask (Cytoplasm) |
| Marburg virus (Angola) | mm5D7 anti-GP |  |  |

Fig S1: Analysis of RDV+favipiravir combination in an anti-MARV cell-based assay using SynergyFinder.

1. Does-response curve for RDV and combination data matrix


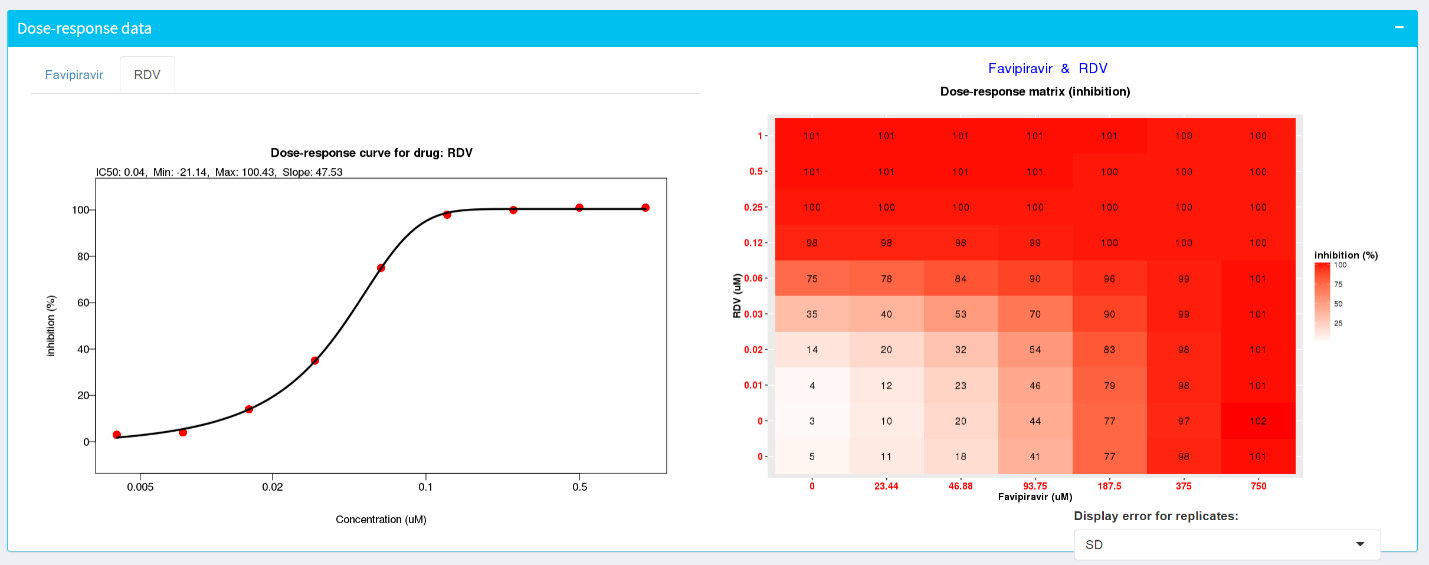


1. Does-response curve for favipiravir and combination data matrix


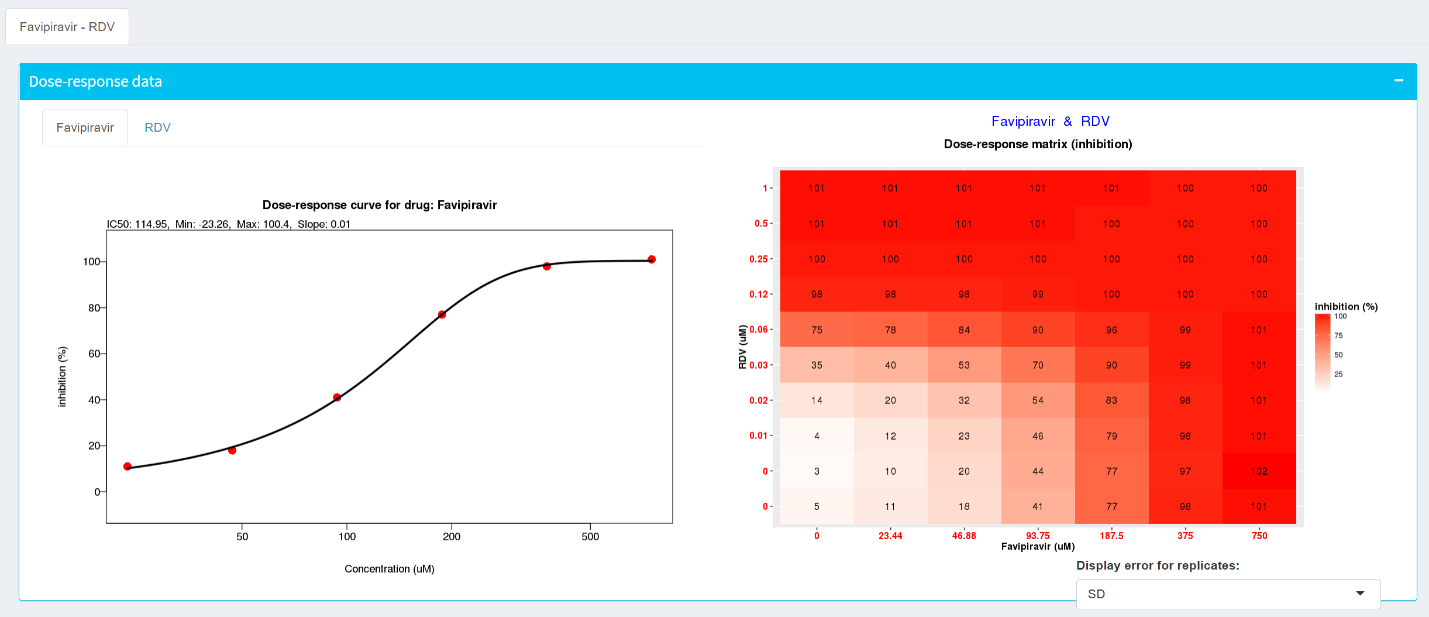


1. Synergy score of 1.965 and synergy map


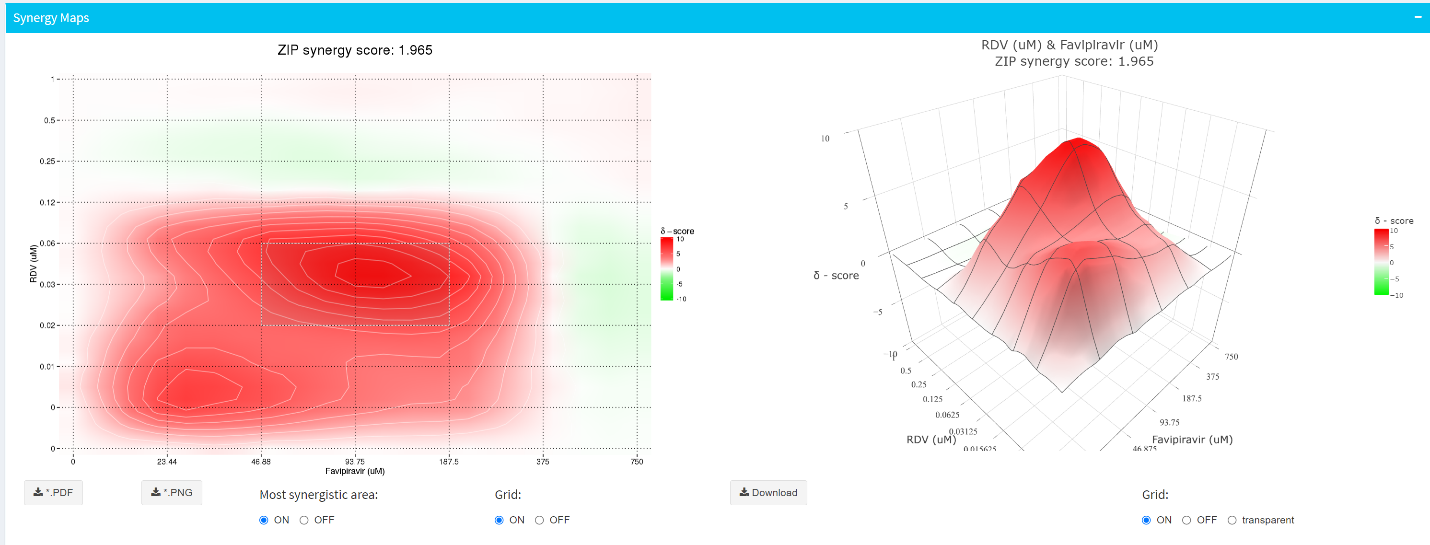

Supplement: Supplementary file 1 — Supplementary Information. [file 41598_2023_29517_MOESM1_ESM.docx]
